# Effects of cooling vest and personal protective equipment removal on thermoregulation in wildland firefighters during progressive thermal loads

**DOI:** 10.3389/fpubh.2024.1408591

**Published:** 2024-08-07

**Authors:** Jorge Gutiérrez-Arroyo, Jose A. Rodríguez-Marroyo, Fabio García-Heras, Juan Rodríguez-Medina, Pilar S. Collado, José G. Villa-Vicente, Belén Carballo-Leyenda

**Affiliations:** VALFIS Research Group, Department of Physical Education and Sports, Institute of Biomedicine (IBIOMED), Universidad de León, León, Spain

**Keywords:** active cooling, passive cooling, thermoregulation, heat strain mitigation, personal protective equipment

## Abstract

**Background:**

Wildland firefighters (WFFs) regularly face demanding physical and environmental conditions during their duties, such as high ambient temperatures, challenging terrains, heavy equipment and protective gear. These conditions can strain thermoregulatory responses, leading to increased fatigue and posing risks to their health and safety. This study examined the effectiveness of two cooling interventions during physical activity in hot environments.

**Methods:**

Eight active male WFFs participated, comparing the effects of wearing a cooling vest (VEST) and personal protective equipment removal (PASSIVE) against a control condition (PPE). Participants walked on a treadmill at a speed of 6 km·h^−1^ for approximately 75-min under hot conditions (30°C and 30% relative humidity). Incremental slope increases were introduced every 15 min after the initial 20 min of activity, with 5-min passive recovery between each increment. Physiological and perceptual parameters were monitored throughout the protocol.

**Results:**

Significant main effects (*p* < 0.05) were observed in skin temperature (36.3 ± 0.2, 36.2 ± 0.4 and 35.4 ± 0.6°C in PPE, PASSIVE and VEST, respectively), physiological strain index (5.2 ± 0.4, 5.6 ± 1.1 and 4.3 ± 1.4 in PPE, PASSIVE and VEST) and thermal sensation (6.6 ± 0.6, 6.4 ± 0.7 and 5.3 ± 0.7 in PPE, PASSIVE, and VEST). However, no significant effects of the cooling strategies were observed on heart rate, gastrointestinal temperature or performance.

**Conclusion:**

Despite the observed effects on physiological responses, neither cooling strategy effectively mitigated thermal strain in WFFs under the experimental conditions tested.

## 1 Introduction

Wildland firefighting entails demanding physical activity over extended periods (1). These challenges are exacerbated by contextual factors such as high ambient temperatures, heavy manual tool handling, and steep terrain, all while wearing personal protective equipment (PPE) (2, 3). However, the cumulative effect of these factors can lead to a non-compensable heat load (3, 4), compromising physical performance and increasing the risk of heat-related disorders or even heatstroke (5, 6). To mitigate these risks, various cooling strategies have been explored, including personal cooling garments (7–9) and passive cooling techniques (10).

The scientific literature has extensively investigated the efficacy of personal cooling garments, such as water-perfused suits, air-perfused suits, ice vests, and phase change material vests, in mitigating heat strain and extending working durations in thermally demanding environments among various groups, including military personnel (11), military firefighters (12), and structural firefighters (8, 13). These garments have been shown to alleviate physiological and perceptual strain by facilitating heat transfer from the body’s core to the periphery, consequently attenuating the rise in central temperature (14). Given that a substantial body surface area is required for effective heat transfer, cooling garments typically focus on the torso, which constitutes approximately 24% of the total body surface area (15). Cooling vests, a subset of personal cooling garments, employ active cooling mechanisms to lower skin temperature through heat conduction (16). Extensively studied in sports contexts (17, 18), cooling vests have demonstrated their capacity to mitigate core temperature elevation and enhance performance when utilized before (pre-cooling) or during exercise (per-cooling), as well as expedite recovery post-exercise (post-cooling) (18). In occupational settings, employing an ice vest or a phase change material vest under firefighter gear (7, 19) and chemical, biological, radiological, and nuclear (CBRN) suits (8, 20) has been found to alleviate physiological and subjective strain during strenuous work in hot conditions, potentially extending tolerance times by 10–20%.

While PPE aims to shield first responders from diverse hazards, it can exacerbate metabolic and thermal responses, especially in hot environments (21, 22). Studies have showed that components such as helmets, gloves, and boots significantly impede body heat dissipation and heighten the thermophysiological strain on wildland firefighters (WFFs) (3) and structural firefighters (22). Consequently, removing one or more PPE components has been proposed as a heat mitigation strategy in occupational literature (10, 23). This passive approach enhances body heat dissipation by facilitating sweat evaporation and improving heat transfer through radiation and convection, thereby reducing body temperature and enhancing perceived comfort (24). Notably, this strategy has been observed to reduce thermal strain to levels comparable to active cooling during breaks in moderate environments (24°C) (10), making it a common choice among structural firefighters and WFFs (9, 25).

Despite the critical impact of heat strain on WFFs’ health and safety, there is a paucity of research investigating the effectiveness of cooling strategies, both active and passive, in mitigating the thermal load experienced by WFFs. This gap leaves unclear whether employing cooling strategies during wildland fire suppression task effectively mitigates the adverse effects of prolonged exposure to high temperatures. It is also crucial to note that while improving aerobic fitness and heat acclimatization have proven to be efficient strategies to combat heat stress over time (26, 27), they require long-term planning to realize benefits. Therefore, alternative measures or strategies may offer more immediate effectiveness. The aim of this study was to address this gap by examining the impact of both active (cooling vest) and passive cooling interventions (PPE removal) on WFFs’ physiological strain. We hypothesized that employing either a cooling vest or removing PPE would enhance heat dissipation in WFFs, leading to decreased core temperature and reduced cardiovascular strain, potentially increasing tolerance time.

## 2 Materials and methods

### 2.1 Subjects

Eight active and healthy male wildland firefighters (mean ± SD; age, 28.8 ± 3.2 years; height, 1.77 ± 0.06 m; body mass, 79.0 ± 17.7 kg; VO_2max_, 51.8 ± 7.8 mL·kg^−1^ min^−1^; body fat, 10.4 ± 3.6%; body surface area, 2.0 ± 0.2 m^2^), with more than 4 years of experience in wildland firefighting, participated voluntarily in this study. They engaged in physical activity 3–4 times per week (45–60 min per training session). None of the subjects reported using saunas or engaging in hot water bathing during the months preceding the study. Informed written consent was obtained from the volunteers before commencing the study. Prior to the commencement of the study, informed written consent was obtained from all volunteers. The study protocol adhered to the guidelines of the Helsinki Conference for research involving human subjects and was approved by the Ethics Committee of the University of León, Spain (025-2020, 22 July 2020).

### 2.2 Experimental design

Each participant completed four trials across four separate testing sessions in September, conducted at the end of the wildfire season. These trials were separated by at least 48 h (48–54 h), during which participants were instructed to avoid strenuous exercise, as well as abstain from consuming alcohol and caffeine within the preceding 24 h. During the initial session, participants’ height, nude body mass and body fat were measured before undertaking a graded exercise test to determine their maximal aerobic power. In the subsequent three trials, participants completed an extended graded heat tolerance test under three experimental conditions following a balanced randomized design: (i) wearing the PPE with no option to remove any part of the equipment or open the personal protective clothing during the test, serving as the control condition; (ii) wearing a cooling vest (Lightweight Body Cooling Vest, Artic Heat^®^, Miami, Australia) underneath the PPE and over the underwear to avoid skin irritation due to prolonged contact (28) (VEST); and (iii) removing the upper part of the protective clothing (i.e., fire-resistant overall), gloves, neck protection and helmet and remaining seated in the hot environmental conditions (i.e., 30°C and 30% relative humidity) during the 5-min recovery periods (PASSIVE).

The PPE utilized by Spanish WFFs (∼6 kg) includes personal protective clothing (65% fire-resistant viscose, 30% Nomex, and 5% Kevlar, surface mass 270 g·m^−2^, thermal resistance 0.019 m^2^ K·W^−1^, evaporative resistance 3.79 m^2^·Pa·W^−1^) and other elements such as helmet, neck protector, gloves, goggles, and mid-calf leather boots. Throughout the different experimental conditions, participants wore the same underwear clothing (i.e., cotton T-shirt, underpants, and socks) under the PPE. The cooling vest (1655.0 ± 485.7 g) was pre-cooled overnight in a freezer at −30°C and placed on each subject just before the trial commenced, positioned over the cotton T-shirt and under the PPE. During the three experimental condition trials, participants carried a backpack (20 kg) simulating the water backpack pump routinely used by Spanish WFFs during fire suppression (2). The total mass of the full ensemble was 27.6 ± 1.8, 26.9 ± 2.1, and 29.4 ± 2.3 kg for the PPE, PASSIVE, and VEST condition, respectively.

### 2.3 Laboratory testing

All tests were performed on a treadmill (h/p Cosmos Pulsar, h/p Cosmos sports and medical GMBH, Nussdorf-Traunstein, Germany). Prior to each tests, participants completed a 10-min warm-up at 60% of maximum heart rate (HR) (8–10 km·h^−1^) followed by 5-min of stretching. The first test conducted was the graded exercise test. During this test, participants wore sportswear (cotton T-shirt, shorts, and sneakers). The test started at a speed of 6 km·h^−1^ was raised by 1 km·h^−1^ every minute until volitional exhaustion. Throughout the entire test, the treadmill slope remained at 1%.

**Figure 1:**
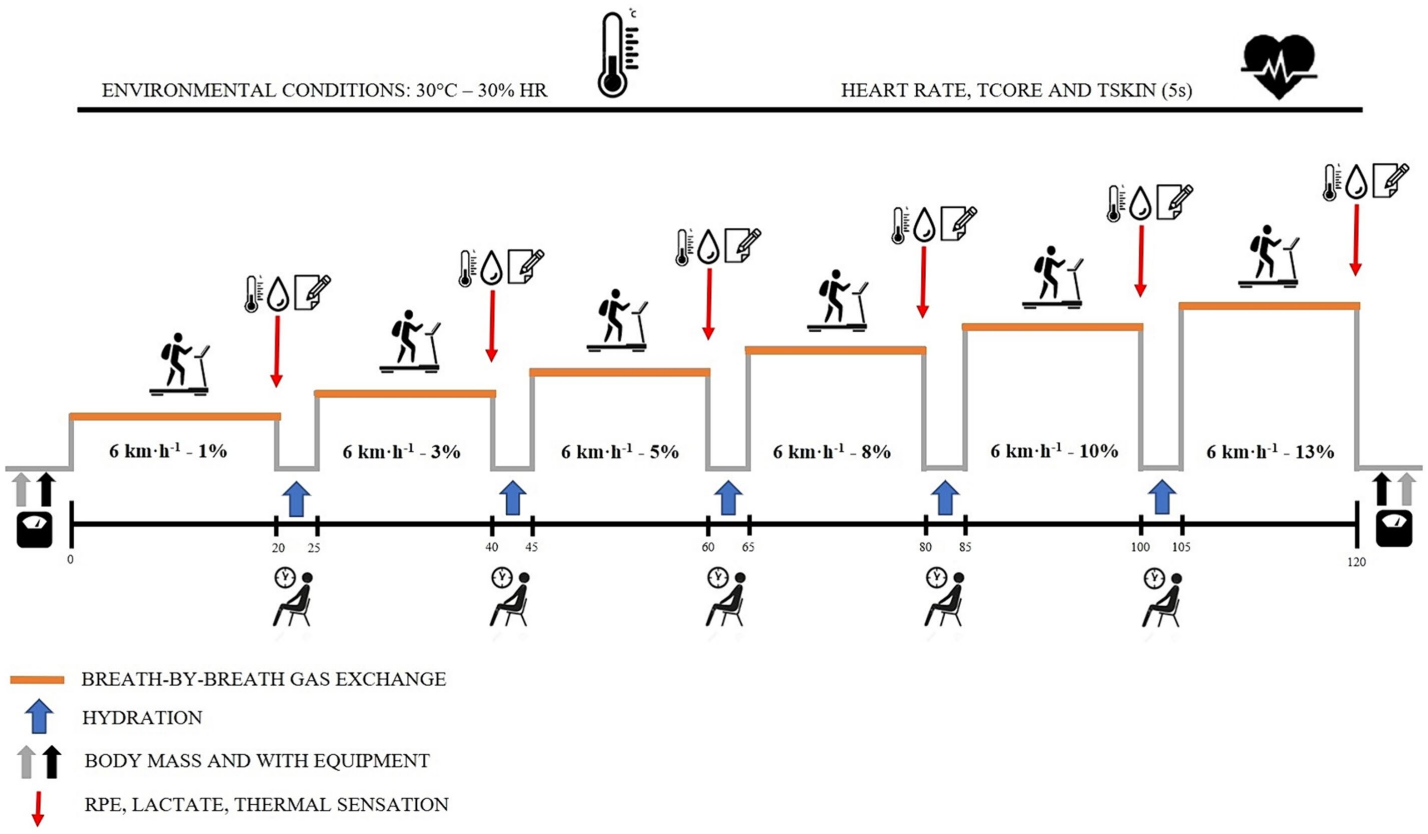
Experimental study protocol overview.

The extended graded heat tolerance tests were conducted at the same time of day (between 09:00 and 13:00 h) under controlled environmental conditions. The air temperature and relative humidity were maintained at 30°C and 30%, respectively, both during exercise and rest, simulating conditions observed during real wildland fire suppressions (2). The experimental protocol involved six bouts of walking at a speed of 6 km·h^−1^ with a gradual increase in slope (1, 2, 5, 8, 10, and 13%), interspersed with passive recovery periods of 5-min in between. The duration of each walking bout was 15-min, except for the first one, which lasted 20-min (Figure 1). This protocol, adapted from previous studies (3, 26), was designed to ensure that the selected speed and slope elicited an exercise intensity greater than 70% of maximum HR. This intensity simulates the moderate to high work intensities typically encountered in real wildland firefighting scenarios (2). During the recovery periods, participants were allowed to drink water *ad-libitum* at a controlled temperature of 15°C (29). The volume of water consumed at the end of each rest period was recorded.

Standard termination criteria were applied during each trial, which included: (i) core body temperature > 39.0°C; (ii) completion of 120-min of work, (iii) reaching 95% of participant’s maximum HR, or (iv) experiencing fatigue, nausea or self-termination (29).

### 2.4 Measurements

During all experiments, heart rate (HR) response and respiratory gas exchange were continuously monitored using a 12-lead electrocardiogram and a breath-by-breath system, respectively (Medisoft Ergocard Professional, Medisoft Group, Sorinnes, Belgium). The maximum oxygen consumption (VO_2max_) was determined as the highest 30-s moving average. HR and VO_2_ data from the final 5 min of each exercise stage were considered as representative measurements for the extended graded heat tolerance test.

Body core temperature was recorded throughout the experimental trials using a gastrointestinal temperature pill (T_gi_) (e-Celsius^®^ Performance, Bodycap, Hérouville Saint-Clair, France), which participants activated and swallowed at least 8 h before commencing the test (30). Skin temperature (T_skin_) was measured using four iButton sensors (DS1922L-F50, Maxim Integrated, Sunnyvale, California, United States), attached to the left major pectoral, medial left triceps, left anterior thigh and left calf with surgical tape (Fixomull, BSN Medical, Hamburg, Germany). The mean T_skin_ was calculated using the equation proposed by Ramanathan (31). Similarly, T_gi_ and T_skin_ were recorded every minute, with their values from the last 5 min of each exercise stage used for analysis.

The Physiological Strain Index (PSI) was calculated using T_gi_ and HR at baseline and every minute according to Tikuisis et al. (32). The PSI values were averaged over the final 5 min of each exercise stage for analysis. Capillary blood samples were collected from the earlobe after the completion of each exercise bout to measure blood lactate concentration (Lactate Scout, Senslab, Leipzig, Germany). During the final 30 s of each exercise stage, the Rating of Perceived Exertion (RPE) was assessed using the Borg scale (0–10) (33), administered consistently by the same researcher. Participants’ thermal sensation was recorded at the end of each exercise bout using a categorical scale (0–8) (34), with verbal anchors representing varying degrees of temperature sensation. Additionally, participants’ whole-body wet sensation was recorded at the end of each trial using a categorical scale (1–9) (35), with verbal anchors representing different levels of wetness.

At the beginning and end of each trial, participants, dressed only in underwear, and each clothing component was individually weighed (50 K150, COBOS, Hospitalet de Llobregat, Barcelona, Spain). This allowed for the calculation of total sweat production, sweat evaporation, and sweat residue in clothing and equipment (35, 36). Total sweat was adjusted for fluid intake. Finally, sweat efficiency was calculated as the ratio between sweat evaporation and total sweat (35, 36). Prior to the trial, urine specific gravity (USG) was measured with a refractometer (Atago URICON-NE, Atago, Tokyo, Japan) to confirm euhydration (USG ≤ 1.020). Participants ingested a bolus of water equivalent to 5 mL·kg^−1^ of body mass 30 min before the test to minimize the risk of dehydration (4).

Body heat storage was calculated as (ΔT_b_ × Δt^−1^) × BM × A_D_^−1^ × c_p_, where cp represented the specific heat of body tissue (3,480 J·kg^−1^°C^−1^) and BM is the body mass in kg (37). Mean body temperature (T_b_) in °C was estimated as T_b_ = 0.8·T_gi_ + 0.2·T_skin_, as recommended for warm environments (37). The rate of change of T_b_ over the duration of the test (seconds) was calculated as ΔT_b_ × Δt^−1^ in °C·s^−1^.

### 2.5 Statistical analyses

The results are presented as mean ± standard deviation (SD). Normality assumption was assessed using the Shapiro–Wilk’s test. The variables analyzed throughout trials were compared using a repeated measures two-way ANOVA with two within-subject factors (experimental condition and time). A one-way ANOVA with repeated measures was used to assess differences between body heat storage and sweat parameters. Bonferroni’s test was employed to determine significant differences between means when a significant *F*-value was obtained. Sphericity assumption was checked using Mauchly’s test; in cases of violation, the Greenhouse–Geisser adjustment was applied. For ordinal variables such as RPE, thermal and wet sensation, the Friedman test for repeated measures was employed to compare these variables across conditions and time. *Post hoc* analyses were conducted using the Wilcoxon signed-rank test with Bonferroni correction. Effect size was calculated using partial eta-squared (η_p_^2^) for dependent variables, with values of 0.01, 0.06 and 0.14 interpreted as small, moderate and large effect sizes, respectively (38). Additionally, the magnitude of differences in pairwise analysis between experimental conditions was expressed as a standardized mean difference using Cohen’s *d*, with values of <0.20, 0.20–0.50, 0.51–0.80, and >0.80 categorized as trivial, small, moderate and large effects, respectively (39). Statistical significance was set at *p* < 0.05. All statistical analyses were performed using SPSS V.27.0 statistical software (SPSS, Inc., Chicago, IL, United States).

## 3 Results

**Figure 2:**
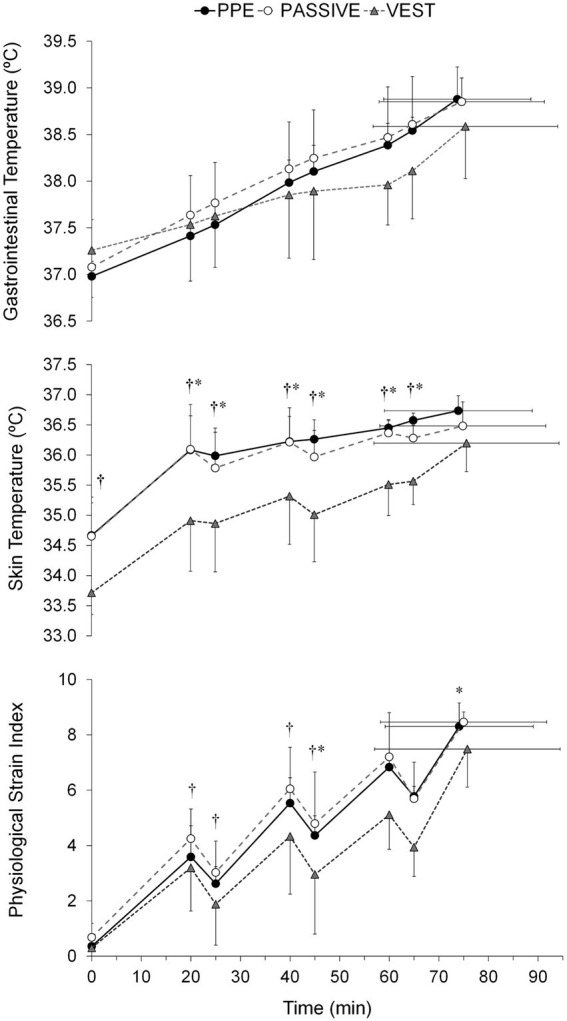
Gastrointestinal and mean skin temperatures alongside physiological strain index across trials: personal protective equipment (PPE), passive cooling (PASSIVE) and cooling vest (VEST) conditions. Values are means ± SD. †, differences between PASSIVE and VEST (*p* < 0.05). *, differences between PPE and VEST (*p* < 0.05).

**Figure 3:**
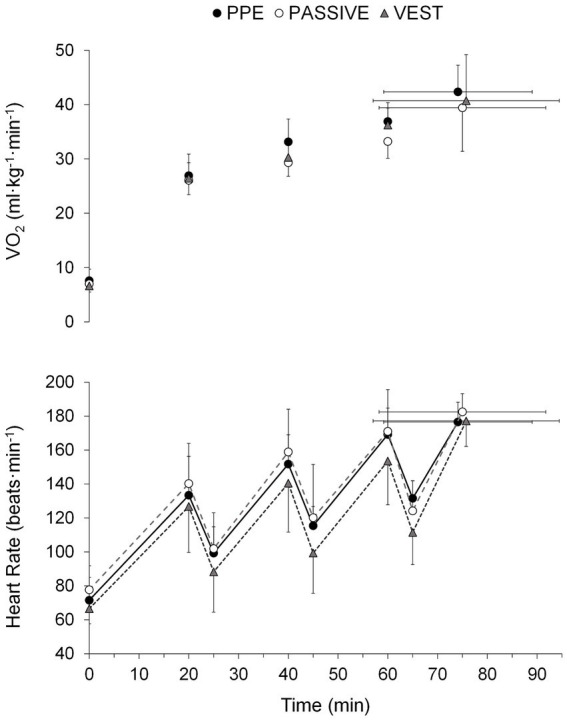
Comparison of oxygen consumption (VO_2_) and heart rate across trials under personal protective equipment (PPE), passive cooling (PASSIVE) and cooling vest (VEST) conditions. Values are means ± SD.

**Figure 4:**
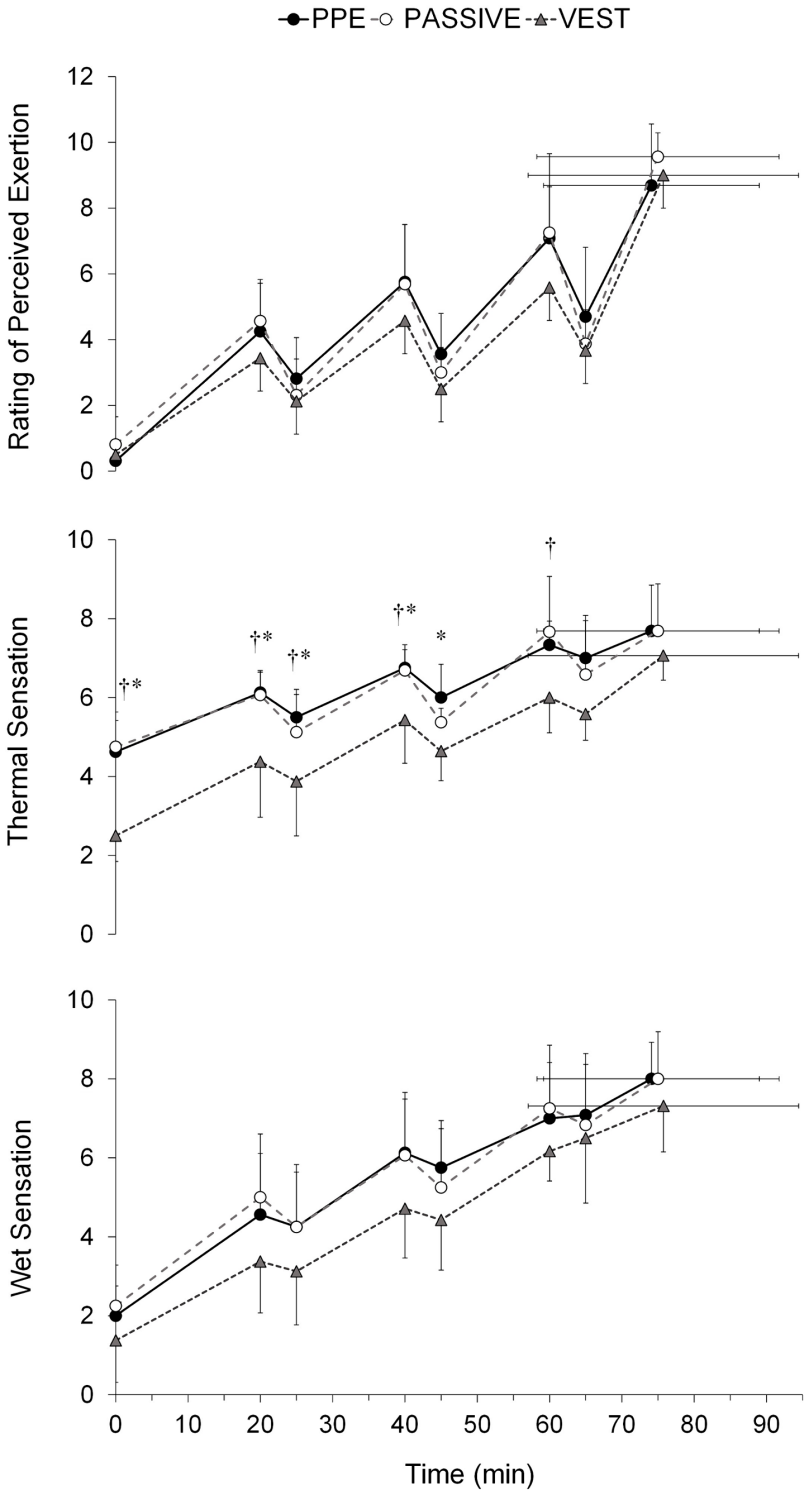
Perceived exertion, thermal sensation and humidity sensation across trials under personal protective equipment (PPE), passive cooling (PASSIVE) and cooling vest (VEST) conditions. Values are means ± SD. †, differences between PASSIVE and VEST (*p* < 0.05). *, differences between PPE and VEST (*p* < 0.05).

Tolerance time was similar across all conditions (74.1 ± 14.9, 75.0 ± 16.7, and 75.7 ± 18.7 min in PPE, PASSIVE, and VEST, respectively). However, only T_skin_ (*F* = 12.9, *p* < 0.01, η_p_^2^ = 0.62), PSI (*F* = 6.0, *p* < 0.05, η_p_^2^ = 0.50) RPE (χ^2^ = 9.3, *p* < 0.05, η_p_^2^ = 0.47) and thermal sensation (χ^2^ = 12.3, *p* < 0.01, η_p_^2^ = 0.60) were significantly affected by the study conditions. Lower (p < 0.05) T_skin_, RPE, thermal and wet sensation were observed in the VEST condition (Table 1). Notably, significant differences (*p* < 0.05) in PSI were found only between the PASSIVE and VEST conditions. Furthermore, the thermophysiological response of the subjects varied with the test time (*F* = 26.9–325.3, χ^2^ = 77.4–101.3, *p* < 0.001, η_p_^2^ = 0.61–0.96) (Figures 2–4). A significant interaction (*p* < 0.05) was observed between the study condition and test time in T_skin_ (*F* = 2.8, *p* < 0.05, η_p_^2^ = 0.32) and PSI (*F* = 2.5, *p* < 0.05, η_p_^2^ = 0.30).

**Table 1:** Physiological values (mean ± SD) recorded during the extended graded heat tolerance test under personal protective equipment (PPE), passive cooling (PASSIVE), and cooling vest (VEST) conditions.

	PPE	PASSIVE	VEST	Standardized mean differences (95% CI)		
				PPE vs. PASSIVE	PPE vs. VEST	PASSIVE vs. VEST
Oxygen consumption (ml·kg^−1^·min^−1^)	34.6 ± 3.0	31.8 ± 2.7	33.2 ± 4.2	0.98 (−0.10, 1.96)	0.38 (−0.63, 1.35)	−0.40 (−1.36, 0.61)
Oxygen consumption (% VO_2max_)	68.2 ± 10.4	62.1 ± 6.0	64.6 ± 4.5	0.72 (−0.33, 1.69)	0.45 (−0.57, 1.42)	−0.47 (−1.44, 0.55)
Heart rate (bpm)	139 ± 13	143 ± 21	131 ± 22	−0.23 (−1.20, 0.77)	0.44 (−0.57, 1.41)	0.56 (−0.47, 1.52)
Heart rate (% HR_max_)	73.7 ± 5.7	75.8 ± 9.3	69.4 ± 9.7	−0.27 (−1.24, 0.73)	0.54 (−0.48, 1.51)	0.67 (−0.37, 1.64)
Blood lactate concentration (mmol·l^−1^)	3.4 ± 1.1	3.7 ± 2.2	4.1 ± 1.8	−0.17 (−1.14, 0.82)	−0.47 (−1.44, 0.55)	−0.20 (−1.17, 0.79)
Gastrointestinal temperature (°C)	38.1 ± 0.1	38.2 ± 0.4	38.0 ± 0.5	−0.34 (−1.31, 0.66)	0.28 (−0.72, 1.25)	0.44 (−0.57, 1.41)
Skin temperature (°C)	36.3 ± 0.2	36.2 ± 0.4	35.4 ± 0.6*†	0.32 (−0.69, 1.28)	2.01 (0.72, 3.08)	1.57 (0.38, 2.58)
Physiological strain index	5.2 ± 0.4	5.6 ± 1.1	4.3 ± 1.4†	−0.48 (−1.45, 0.54)	0.87 (−0.19, 1.85)	1.03 (−0.06, 2.01)
Rating of perceived exertion	5.3 ± 1.1	5.2 ± 1.0	4.4 ± 0.9*†	0.09 (−0.89, 1.07)	0.90 (−0.18, 1.87)	0.84 (−0.22, 1.81)
Thermal sensation	6.6 ± 0.6	6.4 ± 0.7	5.3 ± 0.7*†	0.31 (−0.70, 1.28)	1.99 (0.71, 3.06)	1.57 (0.38, 2.59)
Wet sensation	6.0 ± 1.1	6.0 ± 1.3	4.9 ± 0.9*	0.00 (−0.98, 0.98)	1.09 (0.01, 2.08)	0.98 (−0.10, 1.96)

% VO_2max_, percentage of the maximal oxygen uptake; % HR_max_, percentage of the maximal heart rate. The standardized mean differences were calculated using Cohen’s d and are reported along with the 95% confidence intervals (CI). *, differences with PPE (*p* < 0.05). †, differences with PASSIVE (*p* < 0.05).

Finally, the body heat storage (58.4 ± 7.6, 52.7 ± 9.7, and 45.3 ± 15.0 W·m^2^), fluid intake (855.0 ± 345.5, 750.0 ± 523.8, and 738.7 ± 365.3 mL), total sweat loss (1922.7 ± 511.3, 1895.0 ± 578.5, and 1585.0 ± 502.4 gr), sweat rate (1,317 ± 363, 1,413 ± 457, and 1,229 ± 349 g·h^−1^) and evaporative efficiency (49.5 ± 4.7, 46.3 ± 13.1, and 46.5 ± 14.6%) were similar between PPE, PASSIVE, and VEST conditions.

## 4 Discussion

The present study aimed to assess the impact of wearing a cooling vest or removing the top part of the PPE ensemble during intermittent rest periods on the thermal strain and tolerance time of WFFs. The primary finding of this investigation was that while the cooling vest led to reductions in mean T_skin_, PSI, RPE and thermal sensation throughout most of the test, it did not have a significant effect on exercise tolerance time. However, neither cooling strategy proved effective in reducing the thermal or cardiovascular load or enhancing the tolerance time of WFFs under the experimental conditions tested.

Overall, the results from all three conditions revealed a substantial thermoregulatory and cardiovascular strain, as evidenced by final values of T_gi_ nearing 39°C (38.9 ± 0.4, 38.8 ± 0.3, and 38.6 ± 0.6°C in PPE, PASSIVE and VEST condition, respectively) and average HR values reaching 95% of maximal individual HR (93.8 ± 4.4, 96.9 ± 2.5, and 94.0 ± 4.8% in PPE, PASSIVE and VEST condition, respectively). These findings are consistent with previous laboratory-based studies investigating the physiological response of individuals wearing personal protective equipment (PPE) in hot conditions (3, 21, 22). The combination of environmental conditions and the high metabolic rate induced by the incremental protocol led to an uncompensable heat strain, irrespective of the cooling intervention, resulting in similar metabolic rates, cardiovascular loads (Table 1) and performance durations (~75 min).

The cooling vest did mitigate the increase in T_gi_, particularly in the second half of the trial. However, this effect was insufficient to counter the high thermal load, as indicated by the similar rate of increase in T_gi_ across all conditions (0.025 ± 0.004, 0.023 ± 0.004 and 0.017 ± 0.008°C·min^−1^ in PPE, PASSIVE and VEST condition, respectively). Nevertheless, the lower PSI observed in the VEST condition suggests a beneficial impact of the cooling vest on overall physiological load. This effect may be attributed to the lower T_skin_ (Figure 2) in the VEST condition (PPE vs. VEST = 0.94°C, *p* < 0.05; PASSIVE vs. VEST = 0.79°C, *p* < 0.05) which may have resulted in reduced cutaneous blood flow and cardiovascular effort (40). However, the lack of significant effects of the cooling vest in our study is consistent with the findings of Teunissen et al. (7) and Carter et al. (41), who also examined the impact of cooling vests on structural firefighters’ heat strain during exercise (per-cooling). Specifically, this cooling intervention had no effect on core temperature or HR when firefighters performed steady-state exercise in controlled laboratory conditions (30°C and 50% relative humidity) (7), simulated an intervention in an underground tunnel wearing gas-tight suits, or engaged in a simulated rescue activity in either a hot (170°C) or neutral (15–20°C) environment (41).

While many studies in the literature have reported that cooling vests effectively decrease heat strain and increase tolerance time (8, 19, 20, 42), discrepancies in study protocols, particularly in terms of the net heat load imposed on participants, may explain the differing results. Moreover, the intensity of the cooling treatment might have been insufficient to adequately alleviate the uncompensable heat strain (23). Previous studies utilizing ice vests and phase change material vests with melting points above 10°C have also failed to demonstrate reductions in HR and core temperature (43, 44), indicating the necessity of substantial cooling power to impact core temperature. The effectiveness of cooling vests is largely influenced by factors such as mass, coverage area, and cooling medium (7, 19). Thus, the results obtained suggest that the cooling vest tested might not have had sufficient cooling power to adequately mitigate the thermal stress imposed by the experimental conditions.

During the breaks the passive recovery had a significant effect on T_skin_, but not on T_gi_ or HR, being the cooling effect dissipated over subsequent exercise bouts. This observation may be attributed to the short duration of the breaks and the challenges associated with donning and doffing sweat-soaked PPE (45), which can impede the effectiveness of passive cooling during rest periods (4, 46, 47). Furthermore, the lack of an effect of the passive recovery might be linked to the fact that this strategy was implemented in the same hot environment as the treadmill walking bouts (30°C and 30% relative humidity), which might have limited its efficacy. In contrast to our findings, Hostler et al. (10) found that when performed during 20-min breaks in moderate environments (24°C), passive recovery strategy might reduce thermal strain to the level of active cooling in structural firefighters. These results suggest that for passive recovery to be effective, it should be conducted in thermoneutral environments and for a significantly longer duration than that applied in our study.

The significantly lower thermal sensation observed with the cooling vest compared to the other conditions may be linked to the lower mean T_skin_ achieved in the VEST condition. These findings support previous research indicating that thermal sensation is closely correlated with T_skin_ in warm environments (4, 7). However, it’s worth noting that a low thermal sensation without a concomitant reduction in T_gi_, as observed in the VEST condition, might lead wildland firefighters to perceive a false sense of comfort, potentially exacerbating heat strain and the risk of heat-related illnesses if work continues (4).

No differences were found in total sweat loss or evaporative efficiency between conditions. However, the volume of sweat produced was lower in the VEST condition compared to PPE (~18%) and PASSIVE (~16%). This reduction in sweat loss might be attributed to the visibly lower T_skin_ in the VEST condition (7, 11). Alternatively, the cooling vest might hinder sweat evaporation from the body (43) and fail to contribute positively to the heat balance, as evidenced by the approximately 3% lower evaporative efficiency in the VEST condition compared to the PPE condition. However, the lack of statistically significant differences in total sweat produced and evaporative efficiency across conditions suggest that evaporation may have been equally restricted across conditions by the WFFs’ PPE (7).

The present study had several potential limitations. One of these was the small sample size, which might restrict the study’s conclusions and limit the ability to observe the effectiveness of the cooling strategies examined. Future studies should consider the expected variability in measures of thermoregulation and include a larger sample size (~20 subjects) to improve the robustness of the assessment of the cooling strategies. Additionally, all participants were male WFFs, meaning the results cannot be extrapolated to female WFFs. The different anthropometric characteristics and thermophysiological response of females compared to males (48) could condition the impact of the examined strategies. Another limitation was the hydration protocol used. Participants were allowed to drink water *ad-libitum* during rest periods, which could have been a confounding variable in this study. However, we believe this did not substantially affect the results, as there were no significant differences in the amount of liquid consumed by WFFs under the different conditions. Furthermore, this strategy may be appropriate for ensuring optimal fluid replacement (49). Lastly, the use of the cooling vest resulted in an approximate 2 kg increase in weight compared to the other conditions, which could have increased the workload for the subjects, particularly in the final minutes of the test (~2%) and raised the metabolic cost of walking in the VEST condition, potentially limiting the effectiveness of this strategy. However, since this weight was distributed around the torso, its impact on the metabolic rate was small (50).

In summary, despite the main effects observed in T_skin_, PSI, RPE and thermal sensation due to the study conditions, as well as the interaction effect between conditions and test time on T_skin_ and PSI, these did not translate into an increase in tolerance time or a reduction in core temperature and cardiovascular effort among WFFs. While these findings suggest a more pronounced effect of using cooling vests on WFFs’ responses, the high metabolic rate of the protocol or the duration and environmental conditions during recovery periods might have influenced the results obtained. Therefore, future studies should investigate whether passive cooling strategies, coordinated with appropriate work:recovery protocols, could prove effective. Additionally, although practical limitations may exist regarding the use of cooling vests, further research should explore their potential as per-cooling measures or as a post-fire intervention recovery strategy for WFFs facing high thermal loads.

## Data availability statement

The raw data supporting the conclusions of this article will be made available by the authors, without undue reservation.

## Ethics statement

The studies involving humans were approved by Ethics Committee of the University of León. The studies were conducted in accordance with the local legislation and institutional requirements. The participants provided their written informed consent to participate in this study.

## Author contributions

JG-A: Conceptualization, Data curation, Investigation, Methodology, Writing – original draft. JAR-M: Formal analysis, Funding acquisition, Methodology, Writing – review & editing. FG-H: Data curation, Writing – original draft. JR-M: Data curation, Investigation, Visualization, Writing – original draft. PC: Methodology, Writing – review & editing. JV-C: Conceptualization, Funding acquisition, Methodology, Writing – review & editing. BC-L: Conceptualization, Formal analysis, Methodology, Writing – review & editing.
